# RNA Expression Signatures in Glioblastoma: A Systematic Review of Tumour Biology and Therapeutic Targets

**DOI:** 10.32604/or.2025.070031

**Published:** 2025-10-22

**Authors:** Amber Hassan, Badr Hafiz, Taghreed Alsinani, Rakan Bokhari, Dahlia Mirdad, Awab Tayyib, Alaa Alkhotani, Ahmad Fallata, Iman Mirza, Eyad Faizo, Saleh Baeesa, Huda Alghefari, Maher Kurdi

**Affiliations:** 1 European School of Molecular Medicine, University of Milan, Milan, 20139, Italy; 2 Department of Neurosciences, King Faisal Specialist Hospital and Research Center, Jeddah, 21499, Saudi Arabia; 3 Department of Neurosurgery, King Fahad Hospital, Jeddah, 21196, Saudi Arabia; 4 Department of Surgery, Faculty of Medicine, King Abdulaziz University, Jeddah, 21589, Saudi Arabia; 5 Department of Pathology, Faculty of Medicine, University of Jeddah, Jeddah, 21589, Saudi Arabia; 6 Department of Pathology, College of Medicine, Umm Al-Qura University, Makkah, 21955, Saudi Arabia; 7 Department of Internal Medicine, Faculty of Medicine, University of Tabuk, Tabuk, 71491, Saudi Arabia; 8 Department of Family Medicine, Faculty of Medicine, University of Tabuk, Tabuk, 71491, Saudi Arabia; 9 Department of Surgery, Faculty of Medicine, University of Tabuk, Tabuk, 71491, Saudi Arabia; 10 Department of Neuroscience, Doctor Suliman Fakeeh Hospital, Jeddah, 21461, Saudi Arabia; 11 Department of Pathology, Faculty of Medicine, King Abdulaziz University, Rabigh, 21911, Saudi Arabia

**Keywords:** Glioblastoma, systematic review, gene expression, transcriptomics, tumor microenvironment, precision oncology

## Abstract

**Background:**

Glioblastoma (GBM) remains the most aggressive primary brain tumour in adults, marked by pronounced cellular heterogeneity, diffuse infiltration, and resistance to conventional treatment. In recent years, transcriptomic profiling has provided valuable insights into the molecular mechanisms that govern the progression of glioblastoma. This *systematic review* aims to synthesise the current literature on dysregulated gene expression in GBM, focusing on gene signatures associated with stemness, immune modulation, extracellular matrix remodelling, metabolic adaptation, and therapeutic resistance.

**Methods:**

We conducted a systematic search of PubMed, The Cancer Genome Atlas (TCGA), Chinese Glioma Genome Atlas (CGGA), and the GlioVis portal for studies published between January 2005 and April 2025, limited to English-language reports. Studies were eligible if they included adult glioblastoma tissue or patient-derived datasets and reported gene-level expression or clinical associations. Reviews, commentaries, and studies on non-GBM gliomas were excluded. Screening followed the PRISMA 2020 checklist, with 410 records initially identified, 90 duplicates removed, and 125 studies retained after full-text review. Data were synthesised descriptively, and findings were validated against TCGA/CGGA expression datasets to ensure consistency across cohorts.

**Results:**

We categorised recurrently dysregulated genes by their biological function, including transcription factors (*SOX2*, *ZEB2*), growth factor receptors (*EGFR*, *PDGFRA*), immune-related markers (*PD-L1*, *TAP1*, *B2M*), extracellular matrix regulators (*MMP2*, *LAMC1*, *HAS2*), and metabolic genes (*SLC7A11*, *PRMT5*, *NRF2*). For each group, we examine the functional consequences of transcriptional alterations and their role in driving key glioblastoma phenotypes, including angiogenesis, immunosuppression, invasiveness, and recurrence.

**Conclusion:**

We further discuss the prognostic implications of these gene signatures and evaluate their potential utility in precision medicine, including current clinical trials that target molecular pathways identified through transcriptomic data. This review highlights the power of gene expression profiling to stratify glioblastoma subtypes and improve personalised therapeutic strategies.

## 1 Introduction

High-grade astrocytomas are the most prevalent primary malignant brain tumours, accounting for nearly half of all central nervous system (CNS) gliomas. The classification of these tumours has evolved substantially, particularly following the release of the 2016 World Health Organisation (WHO) 4th edition classification, which introduced the integration of histopathological and molecular features. Before 2016, diagnoses were primarily histological and reliant on immunohistochemistry [1]. The incorporation of molecular diagnostics, such as *Isocitrate Dehydrogenase* (IDH) and Alpha Thalassemia Mental Retardation X (*ATRX*), has enabled the stratification of diffuse malignant astrocytomas into biologically distinct subgroups [2]. In the 2021 WHO classification (5th edition), the concept of *IDH*-mutant grade 4 astrocytoma was formally distinguished from its *IDH*-wildtype counterpart [2]. This distinction emphasises that although both subtypes may share treatment approaches and some molecular similarities, they are biologically and clinically distinct. According to the Consortium to Inform Molecular and Practical Approaches to CNS Tumour Taxonomy Not Official WHO (cIMPACT-NOW) consortium, a definitive diagnosis of WHO grade 4 astrocytoma requires IDH mutation, *ATRX* loss, Tumour Protein-p53 (*TP53*) mutation, and absence of 1p/19q code deletion [3]. Conversely, glioblastomas are defined by a wild-type IDH status, alongside hallmark alterations such as Epidermal Growth Factor Receptor (*EGFR*) amplification, Telomerase Reverse Transcriptase (*TERT*) promoter mutations, and chromosomal abnormalities, including the gain of chromosome 7 and loss of chromosome 10 [4].

Despite these refinements, the genetic landscape of both tumour types remains complex and incompletely characterised. Key mutations affect signalling pathways related to cell growth and survival, particularly the Receptor Tyrosine Kinase (*RTK*)/*Rat* Sarcoma (RS)/Phosphoinositide 3-Kinase (PI3K) pathway, *TP53*, and Retinoblastoma (*RB*). *RTK* genes commonly show amplification, splice variants, or fusions. Examples include *EGFR*, Platelet-Derived Growth Factor Receptor Alpha (*PDGFRA*), Mesenchymal-Epithelial Transition (*MET*) factor, and Fibroblast Growth Factor Receptor 3 (*FGFR3*) [5,6]. Other frequent mutations have also been reported [7]. Although many of these mutations are intrinsic to tumour DNA or RNA, their detection in routine practice is limited due to a lack of standardised platforms. As a result, next-generation sequencing (NGS) of both DNA and RNA has become critical for identifying such alterations [8,9]. Beyond tumour-intrinsic factors, there is an increasing focus on the tumour microenvironment, including blood vessels, tumour-associated macrophages (*TAMs*), and tumour-infiltrating lymphocytes (*TILs*) [10,11]. In *IDH*-wildtype glioblastoma, molecular subgroups have been identified through comprehensive genomic and epigenomic profiling, revealing distinct DNA methylation signatures and expression profiles [11,12].

Despite multimodal treatment, including surgery, radiotherapy, and chemotherapy, the prognosis for patients with WHO grade 4 astrocytomas or glioblastomas remains poor, regardless of *IDH* mutation status. Studies suggest that integrating genomic and epigenomic data could enhance the development of clinically relevant molecular classifiers [12–14]. Epigenetic regulation plays a pivotal role in tumour biology, while global hypomethylation activates oncogenes and contributes to genomic instability [15]. Transcriptomic profiling, including analyses of RNA and messenger RNA (mRNA) expression, has advanced the understanding of glioblastoma and high-grade astrocytomas [16]. These tumours display extensive transcriptional heterogeneity, which drives their aggressive nature and treatment resistance [15]. Techniques such as RNA sequencing (RNA-seq) and single-cell RNA sequencing (scRNA-seq) have enabled detailed mapping of gene expression. Integrated transcriptomic studies have revealed shared transcriptional programs between *IDH*-wildtype glioblastomas and *IDH*-mutant grade 4 astrocytomas, mediated by transcription factors such as the neurofibromatosis 1 (*NF1*) family, which play roles in tumour development and maintenance [17].

Furthermore, unique gene expression patterns between tumour subtypes suggest novel therapeutic targets. These transcriptomic advances hold significant promise for precision oncology, allowing patient stratification based on expression signatures and predicted outcomes. Ongoing research in this area is crucial for translating molecular findings into clinical strategies that enhance prognosis and therapeutic efficacy in high-grade gliomas. Despite numerous advances, there remains a lack of consolidated insight into which genes are consistently dysregulated in glioblastoma (GBM) across studies. Therefore, we conducted a systematic review of transcriptomic studies to identify and functionally classify key genes associated with GBM. This review adheres to PRISMA guidelines and incorporates clinical relevance, prognosis, and therapeutic potential.

## 2 Methods

### 2.1 Data Sources and Search Strategy

This study was designed as a systematic review and gene classification project aimed at identifying, annotating, and functionally categorising genes consistently dysregulated in glioblastoma (GBM). The primary objective was to integrate transcriptomic datasets and peer-reviewed literature to construct a curated panel of GBM-associated genes, organised by biological function, prognostic significance, and therapeutic relevance. A comprehensive literature search was conducted using PubMed with search terms including “glioblastoma,” “gene expression,” “prognostic marker,” “immune modulation,” “stemness,” “epigenetic regulation,” and “metabolism,” combined using Boolean operators.

In parallel, transcriptomic data were retrieved from The Cancer Genome Atlas (TCGA) (https://www.cancer.gov/ccg/research/genome-sequencing/tcga) (accessed on 26 August 2025), Chinese Glioma Genome Atlas (CGGA) (https://www.cgga.org.cn) (accessed on 26 August 2025), and the GlioVis data portal (http://gliovis.bioinfo.cnio.es) in April 2025. Specifically, TCGA GBM RNA-seq datasets (Illumina HiSeq) and CGGA GBM microarray datasets (Agilent and Affymetrix platforms) were obtained as pre-processed, normalised gene-level expression matrices aligned to the GRCh38/hg38 human reference genome. TCGA RNA-seq data in GlioVis are normalised using RNA-Seq by Expectation-Maximisation (RSEM) with upper quartile normalisation. In contrast, CGGA microarray datasets are normalised using the Robust Multi-array Average (RMA) method. Searches were limited to English-language studies published between January 2005 and April 2025 reporting data from human GBM tissues or patient-derived datasets.

### 2.2 Study Selection and Eligibility

Studies were considered eligible if they investigated adult glioblastoma (GBM) and reported gene-level expression data or clinical outcome associations. Only primary research articles involving human tumour samples were included. Exclusion criteria were: (i) studies on non-GBM gliomas, (ii) articles lacking specific gene expression data, and (iii) secondary literature such as reviews or commentaries.

The selection process followed the PRISMA 2020 guidelines, with details documented in the PRISMA checklist (Supplementary Material). Of the 410 records initially identified, 90 were duplicates, 121 were excluded during title and abstract screening, and 125 studies were retained after full-text review.

From these, 112 genes were shortlisted based on their presence in at least two independent studies and confirmation in public datasets (TCGA, CGGA, or GlioVis). Each gene was assigned to a single primary functional category: stemness, proliferation/survival, extracellular matrix (ECM)/invasion, immune modulation, metabolic regulation, or epigenetic control to maintain interpretability and clarity in visualisations. In cases where a gene had multiple biological roles, the primary category was determined by the function most consistently supported across at least two independent studies and most relevant to GBM pathogenesis. Functional assignments were derived from Gene Ontology (GO) annotations, PubMed-indexed literature, Gene Expression Omnibus (GEO) dataset descriptors, and TCGA/CGGA clinical correlation.

### 2.3 Data Extraction, Curation, and Scoring

For each gene, we extracted key information including its name, functional category, expression status (upregulated, downregulated, mutated), prognostic relevance, and translational targetability.

***Expression status determination*:** Genes were classified as upregulated or downregulated by cross-referencing results from at least two independent transcriptomic studies and validating trends against TCGA and CGGA datasets. When dataset-specific statistics were available, differential expression thresholds were defined as |log_2_ fold change| ≥ 1 with a false discovery rate (FDR) < 0.05.

***Mutation annotation:*** Mutational status was not considered an expression category in isolation. Instead, for each reported mutation, we documented whether the mutant form was upregulated, downregulated, or unchanged at the mRNA level. Where available, the biological effect of the mutation was noted, for example, gain-of-function EGFRvIII variants or loss-of-function TP53 alterations, and these details were presented in the supplementary annotation tables.

***Aggression score***: o capture tumour-driving potential, an aggression score was calculated as a composite of three weighted components: (i) transcriptomic overexpression in GBM vs. normal brain (+1 if log_2_FC ≥ 1, FDR < 0.05), (ii) prognostic association (+1 if high expression correlated with shorter survival, −1 if with more prolonged survival), and (iii) functional impact (+1 if consistently implicated in ≥2 independent studies as a driver of proliferation, invasion, angiogenesis, or immune evasion). Scores ranged from −1 to +3, with higher values reflecting more aggressive tumour-associated behaviour.

***Targetability score***: Translational feasibility was assessed using three criteria: (i) availability of pharmacological inhibitors or biologics (+1), (ii) inclusion in active or completed cancer clinical trials (+1), and (iii) evidence of blood–brain barrier (BBB) penetration (+1). Scores ranged from 0 to 3, with higher values indicating stronger therapeutic readiness.

All extracted data were organised into a structured reference matrix to facilitate cross-comparison and visualisation. In cases of conflicting findings, priority was given to results supported by clinical validation or consistent trends across datasets. Visualisations were generated using Python (Matplotlib v3.9.0). The corresponding scripts covering data preprocessing, formatting, and plotting are available from the corresponding author upon request. Figures included functional category distributions, multifunctionality heatmaps, molecular subtype alignments, aggression score rankings, interaction networks, and targetability matrices (Tables A1–A7).

### 2.4 Network Analysis

Protein–protein interaction (PPI) analysis was performed using the STRING database (v12.0) (https://string-db.org) (accessed on 26 August 2025), restricted to validate experimentally and high-confidence predicted interactions with a minimum confidence score of 0.7. Disconnected nodes were removed for clarity. The resulting PPI data were imported into Cytoscape (v3.9.1) for visualisation. Hub genes were identified using degree centrality and betweenness centrality metrics, allowing prioritisation of central regulators such as STAT3 and PD-L1.

### 2.5 Data Validation

Beyond the core 112 genes, additional genes of emerging interest (e.g., *OXCT1-AS1, SRC-1, TRIM8, C7orf31, ELAVL2, Ephrin-B2*) were included if reported in ≥2 independent human studies or validated in functional models between 2023 and 2025. Aggression scores were computed as a composite of expression Z-score (TCGA/CGGA), prognostic association (poorer +1, better −1, context-dependent 0), and functional impact (+1 if involved in core GBM pathways such as invasion or immune modulation). Genes with a score ≥two were classified as highly aggressive; ≤−1 as protective.

Where possible, transcriptomic data were cross-checked against proteomic or immunohistochemical (IHC) validation in TCGA, GlioVis, or published studies to enhance biological confidence.

Due to substantial heterogeneity in study designs, patient cohorts, transcriptomic platforms, and outcome measures among the included studies, a formal meta-analysis with pooled hazard ratios or forest plots was deemed inappropriate to avoid introducing bias. Instead, we performed a descriptive synthesis complemented by quantitative summaries, including frequency distributions of upregulated vs. downregulated genes and the proportion of genes associated with favourable or poor prognosis across datasets.

## 3 Results

### 3.1 Study Selection and Functional Landscape

**Figure 1:**
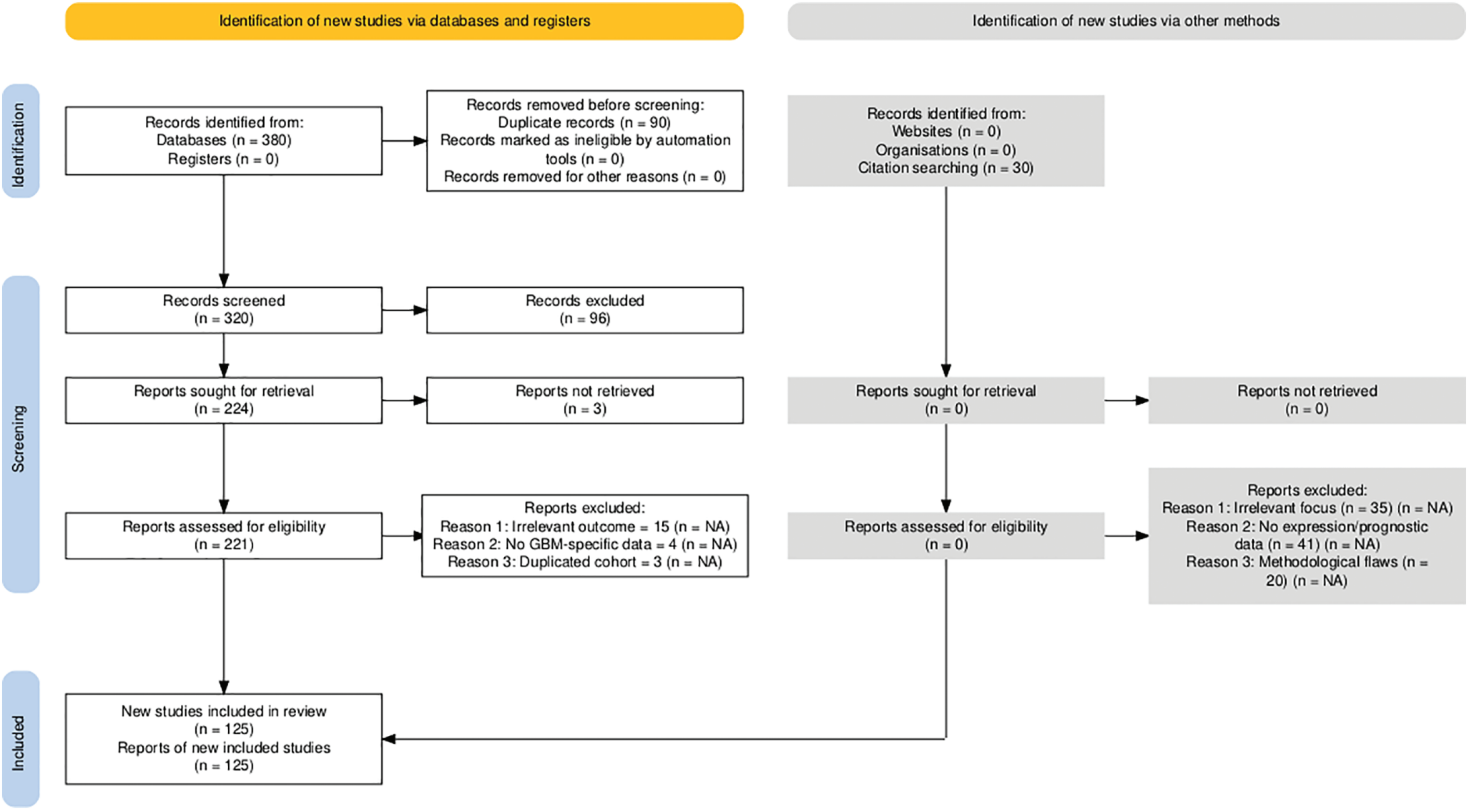
PRISMA 2020 flow diagram detailing the systematic identification, screening, eligibility assessment, and inclusion of studies in the glioblastoma gene expression review. Numbers reflect the sequential filtering from initial records identified (n = 410) through duplicate removal, title/abstract screening, full-text review, and final inclusion (n = 125 studies). NA: Not applicable

**Figure 2:**
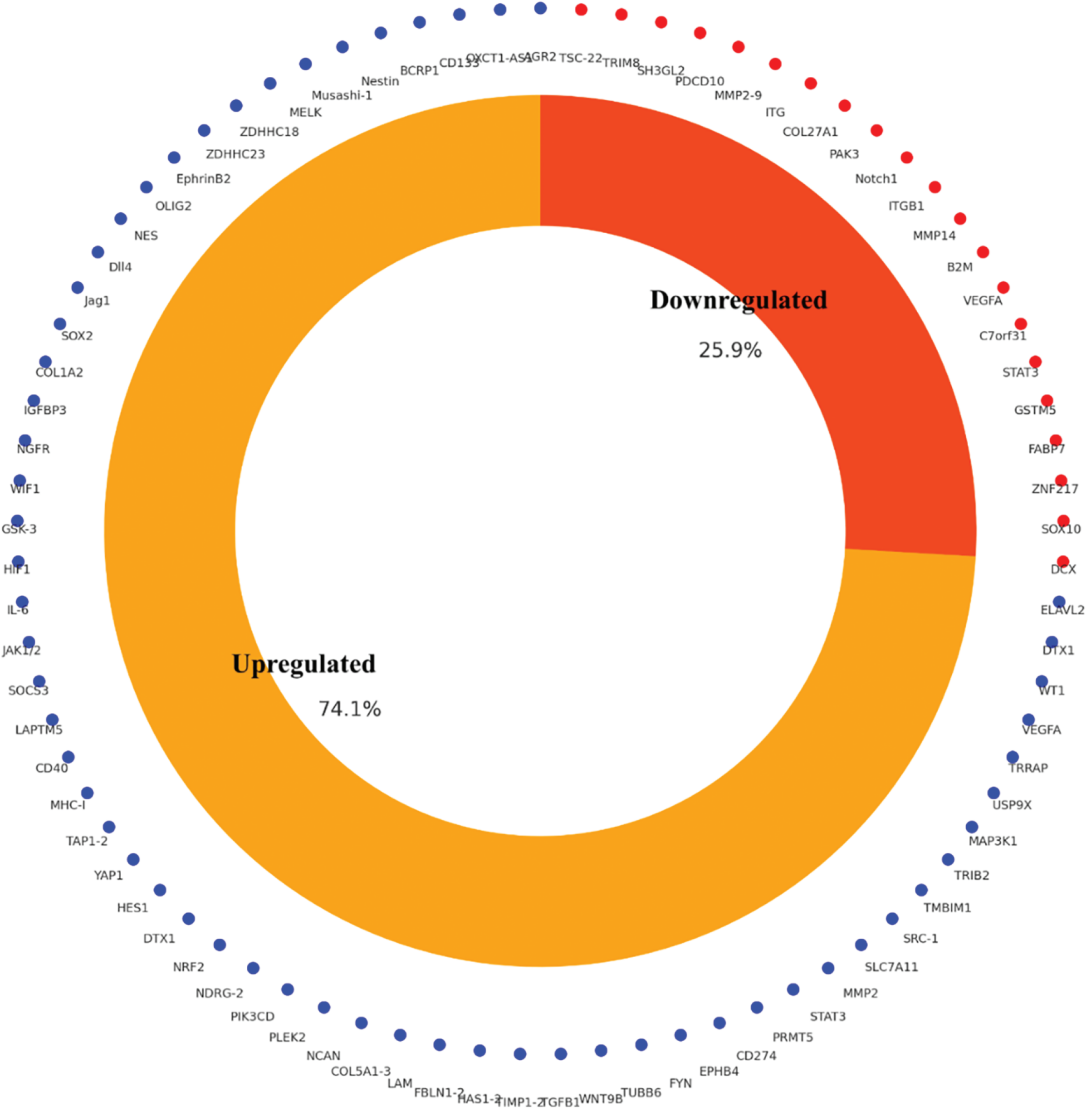
Differential expression profile of glioblastoma-associated genes (n = 85) curated from integrated literature and TCGA/CGGA datasets. A total of 74.1% were consistently upregulated and 25.9% downregulated in GBM relative to non-tumor brain tissue

**Figure 3:**
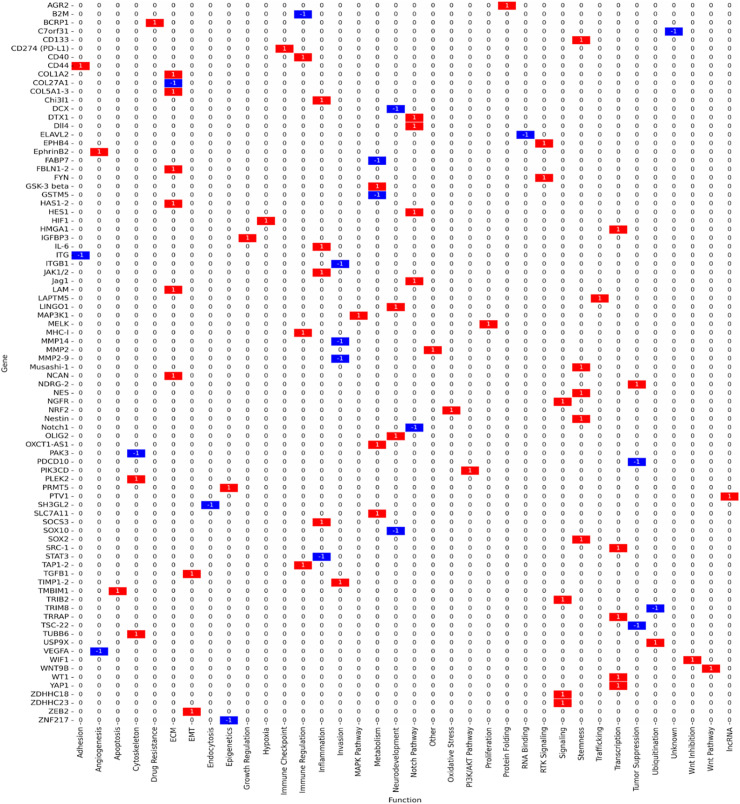
Functional matrix mapping glioblastoma-associated genes across key biological processes: stemness, proliferation/survival, extracellular matrix (ECM)/invasion, immune modulation, metabolic regulation, and epigenetic control. Red bars indicate upregulated genes; blue bars indicate downregulated/tumour suppressors; purple outlines indicate multifunctional genes contributing to more than one biological category. Network relationships were derived using STRING v12.0 (interaction score ≥ 0.7, high confidence) and visualised in Cytoscape v3.9.1, with hub genes prioritised based on node degree and betweenness centrality

The study selection process followed PRISMA 2020 guidelines to ensure transparency and reproducibility. A total of 410 records were identified through database searches (PubMed, TCGA, CGGA, GlioVis) and manual screening. After removal of 90 duplicates and the application of predefined inclusion and exclusion criteria, 125 studies were retained for final synthesis (Fig. 1). Out of 112 curated glioblastoma (GBM)-associated genes identified through systematic review and dataset integration, 85 had complete functional annotations and consistent expression data. These genes formed the basis of quantitative analyses and visualisations. Among these, 74.1% were consistently upregulated and 25.9% were downregulated in glioblastoma (Fig. 2). Functional classification showed that genes involved in extracellular matrix (ECM) remodelling, invasion, stemness maintenance, immune modulation, and metabolic reprogramming were predominant. In addition to functional categorisation, immune modulators (including TAM- and TIL-associated genes) were reviewed for transcript–protein concordance using available proteomic and IHC data. While formal meta-analysis of hazard ratios was not feasible due to heterogeneity in platforms, study designs, and patient cohorts, subgroup-specific survival trends were summarised descriptively, with immune modulators and ECM-remodelling genes showing the strongest association with poor outcomes. The functional matrix (Fig. 3) highlighted multifunctional hubs such as *STAT3*, *TGFB1*, and *PRMT5*, bridging several biological processes central to glioblastoma pathogenesis.

### 3.2 Key Biological Processes in Glioblastoma

Genes linked to stemness and therapy resistance included *CD133* (*PROM1*), *SOX2*, *Nestin*, *OLIG2*, and *Musashi-1*, all of which support the persistence of glioma stem-like cell populations and drive recurrence. These genes were predominantly upregulated in proneural and classical GBM subtypes. *STAT3* functioned as a multifunctional regulator with critical roles in both stemness and immune modulation (Fig. 3, Table 1).

**Table 1:** Summary of functional categories, representative genes, and expression patterns in glioblastoma

Functional category	No. of genes	% Upregulated	Representative Genes
Stemness & therapy resistance	18	89%	CD133 (PROM1), SOX2, Nestin, OLIG2, STAT3
Immune modulation	21	85%	PD-L1 (CD274), IL-6, STAT3, JAK1/2, CD40
Metabolic reprogramming	13	77%	SLC7A11, NRF2 (NFE2L2), PRMT5, MAP3K1
ECM remodelling & invasion	33	91%	MMP2, MMP9, TIMP1, TIMP2, COL5A1, COL5A3, TGFB1
Prognostic outliers*	4	Variable	PAK3, Notch1, B2M, VEGFA

Note: *indicate genes with variable expression patterns that are nevertheless associated with glioblastoma prognosis.

Immune modulators such as *PD-L1* (*CD274*), *IL6*, *B2M*, *TAP1*, *TAP2*, *SOCS3*, *CD40*, and *STAT3* contributed to immune evasion and pro-inflammatory signaling. Approximately 85% of these genes were upregulated, especially in mesenchymal GBM.

Network analysis identified *STAT3*, *PD-L1*, and *JAK1/2* as central immune hubs with translational relevance for immunotherapy (Fig. 3, Table 1). This analysis was performed using the STRING database (v12.0) restricted to experimentally validated and high-confidence predicted interactions (confidence score ≥ 0.7), with visualisation and hub identification conducted in Cytoscape (v3.9.1) based on degree and betweenness centrality metrics.

Prognostic analysis within immune-related genes revealed that high expression of TAM-associated mediators (STAT3, TGFB1, IL10) correlated with reduced survival. In contrast, increased TIL effector signatures (e.g., IFNG, GZMB) were infrequently observed and associated with more prolonged survival in a minority of mesenchymal GBM cases. These patterns align with proteomic observations of M2-polarised macrophage dominance and T-cell exhaustion, reinforcing their value as stratification biomarkers.

Metabolic regulators included *SLC7A11*, *NRF2* (*NFE2L2*), *PRMT5*, *MAP3K1*, and *GSK3B*, which facilitated survival under hypoxia, oxidative stress, and nutrient deprivation. Notably, *SLC7A11* and *PRMT5* were dual regulators of metabolic adaptation and immune evasion, strengthening their therapeutic relevance (Fig. 3, Table 1).

Genes driving ECM remodeling and invasion included *MMP2*, *MMP9*, *TIMP1*, *TIMP2*, *COL5A1*, *COL5A3*, *LAMC1*, *HAS1*, *HAS2*, and *TGFB1*. These genes were consistently upregulated, enriched in mesenchymal GBM, and associated with vascular mimicry, therapy resistance, and poor prognosis.

**Figure 4:**
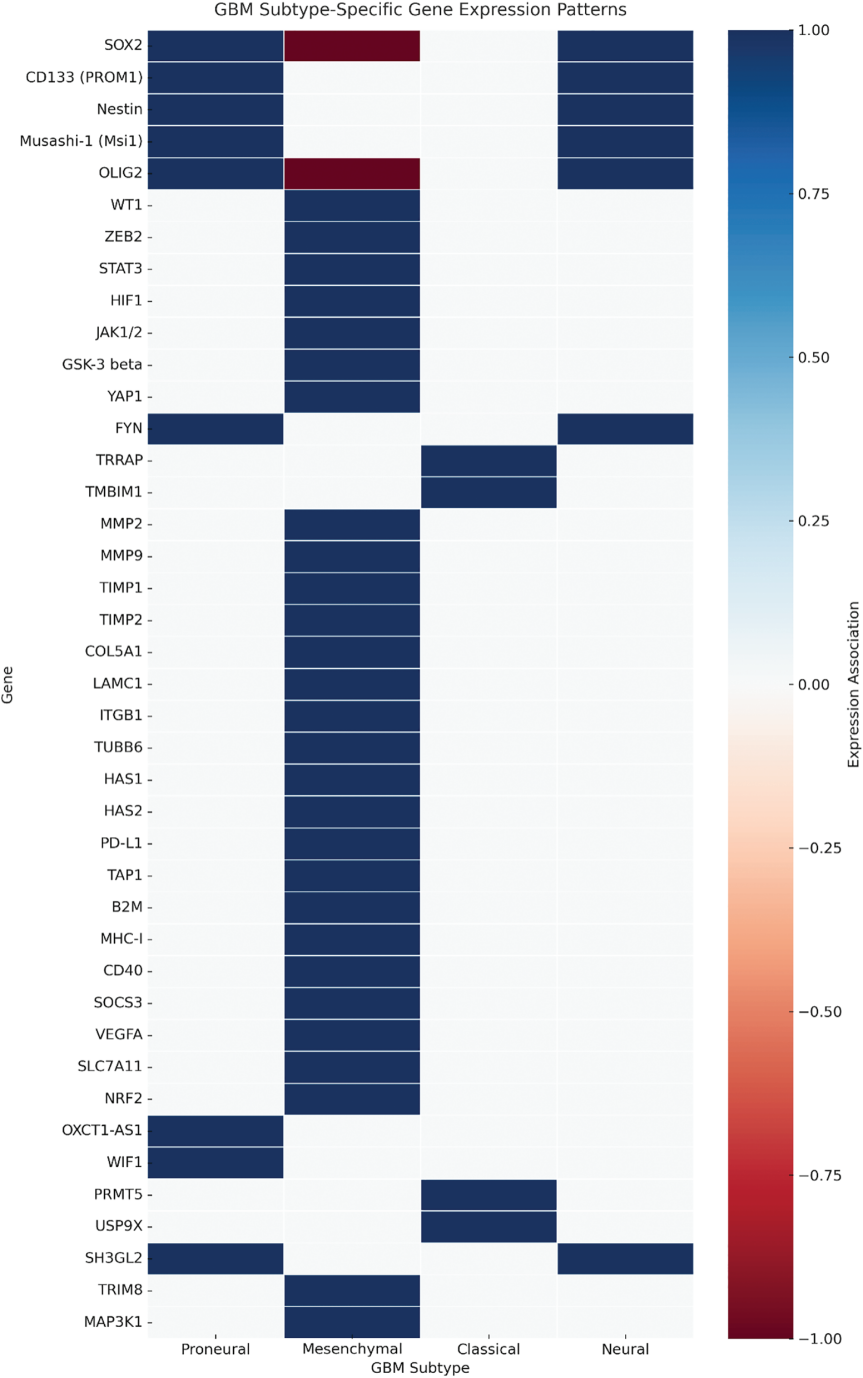
Subtype-specific gene expression heatmap in glioblastoma. Heatmap illustrating relative expression patterns of curated glioblastoma-associated genes across the proneural, mesenchymal, classical, and neural subtypes (TCGA GBM RNA-seq). Genes are grouped by primary functional category: stemness, extracellular matrix (ECM)/invasion, immune modulation, metabolic regulation, proliferation/survival, and epigenetic control. Colour intensity represents Z-score normalised expression. Proneural enrichment is observed for stemness-associated genes such as SOX2, OLIG2, and PROM1; mesenchymal enrichment for ECM and immune modulators, including MMP9, STAT3, and TGFB1; and classical enrichment for proliferation-related genes such as EGFR and CCND2. Neural subtype tumours demonstrate relative enrichment in neuronal lineage markers. Functional categorisation was prioritised based on literature consensus and Gene Ontology annotations

We next examined whether the identified genes exhibited subtype-specific enrichment patterns across the proneural, mesenchymal, classical, and neural glioblastoma subtypes. A subtype heatmap (Fig. 4) was generated, highlighting the preferential expression of stemness-associated genes such as SOX2, OLIG2, and PROM1 in proneural GBM, ECM/invasion and immune modulatory genes such as MMP9, STAT3, and VEGFA in mesenchymal GBM, and cell cycle–linked proliferation genes in classical GBM. Neural subtype tumours demonstrated relative enrichment in neuronal lineage markers.

These subtype-associated expression patterns have direct therapeutic implications: proneural enrichment of OLIG2, SOX2, and PROM1 supports strategies targeting stemness pathways; mesenchymal predominance of MMP9, STAT3, and TGFB1 aligns with anti-invasive and immunomodulatory approaches; and classical subtype enrichment in EGFR and CCND2 suggests sensitivity to EGFR inhibitors or cell cycle-directed agents.

### 3.3 Aggression and Targetability Scoring Classification

Analysis of gene expression patterns and clinical outcomes revealed that 65% of the genes correlated with poor survival, 20% with better prognosis, and 15% demonstrated context-dependent associations. Adverse prognostic markers included *AGR2*, *IL6*, *MMP9*, and *TIMP1*, whereas *PAK3*, *Notch1*, and *B2M* were linked to more prolonged survival or therapeutic responsiveness, underscoring the molecular heterogeneity of GBM. While forest plot visualisation of pooled hazard ratios was considered, methodological heterogeneity in the literature precluded robust statistical synthesis. Instead, subgroup-level prognostic associations (e.g., immune modulators, ECM-remodelling genes) were summarised descriptively, preserving functional granularity while avoiding misleading pooled estimates.

**Figure 5:**
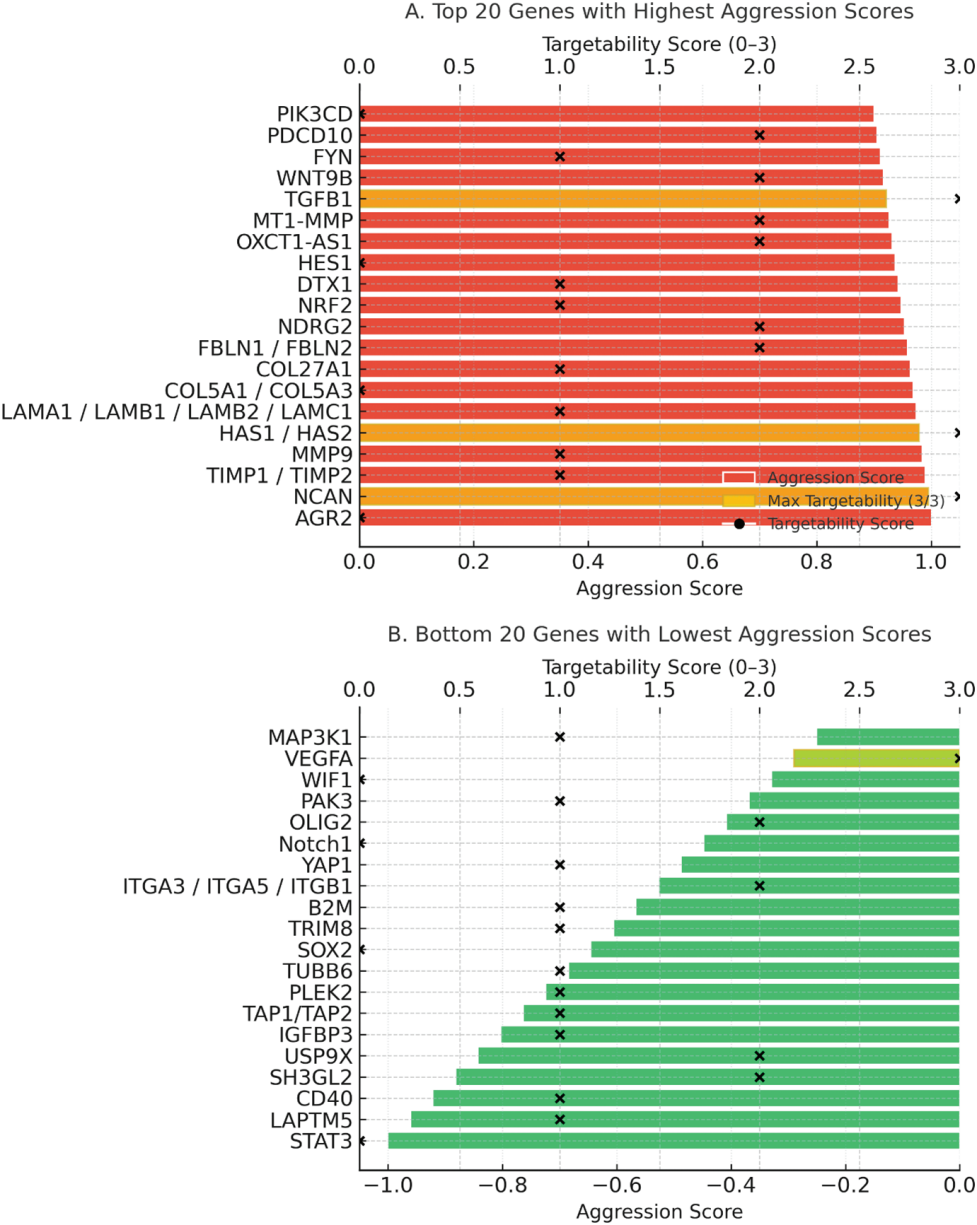
Aggression and targetability scores of glioblastoma-associated genes. (**A**) Twenty genes with the highest aggression scores. (**B**) Twenty genes with the lowest or protective aggression scores. Colored bars represent aggression scores (red = high, green = low). Black “×” symbols indicate the targetability scores (0–3) of each gene, while black dots highlight genes that achieved the maximum targetability score (3/3). Gold highlights within the bars denote genes with maximum targetability, reflecting the presence of available inhibitors, inclusion in clinical trials, and blood–brain barrier penetration

Aggression score analysis integrated expression intensity, prognostic significance, and functional roles. Specifically, aggression scores were calculated as a composite of three weighted components: (i) transcriptomic overexpression in GBM vs. normal brain (+1 if log_2_FC ≥ 1, FDR < 0.05), (ii) prognostic association from survival analysis (+1 if high expression correlated with shorter overall survival, −1 if associated with more prolonged survival), and (iii) functional impact (+1 if implicated in ≥2 independent studies as a driver of core GBM hallmarks such as proliferation, invasion, angiogenesis, or immune evasion). Scores ranged from −1 to +3, with higher values indicating more aggressive tumour-associated behaviour. The top 20 most aggressive genes (Fig. 5, top panel) included *AGR2*, *NCAN*, *MMP9*, *HAS2*, *TIMP1*, *TIMP2*, and *TGFB1*, all of which were strongly associated with ECM remodelling, angiogenesis, invasion, and mesenchymal subtype enrichment. Their high scores (≥+0.9) suggest these genes are core drivers of glioblastoma malignancy. Conversely, the bottom 20 genes (Fig. 5, bottom panel), including VEGFA, ITGA3, ITGA5, ITGB1, Notch1, and PAK3, were linked to protective roles, more prolonged survival, or subtype-specific therapeutic responsiveness. Notably, the negative aggression score of *VEGFA* reflects its predictive role in anti-VEGF therapy rather than an absence of oncogenic potential.

**Figure 6:**
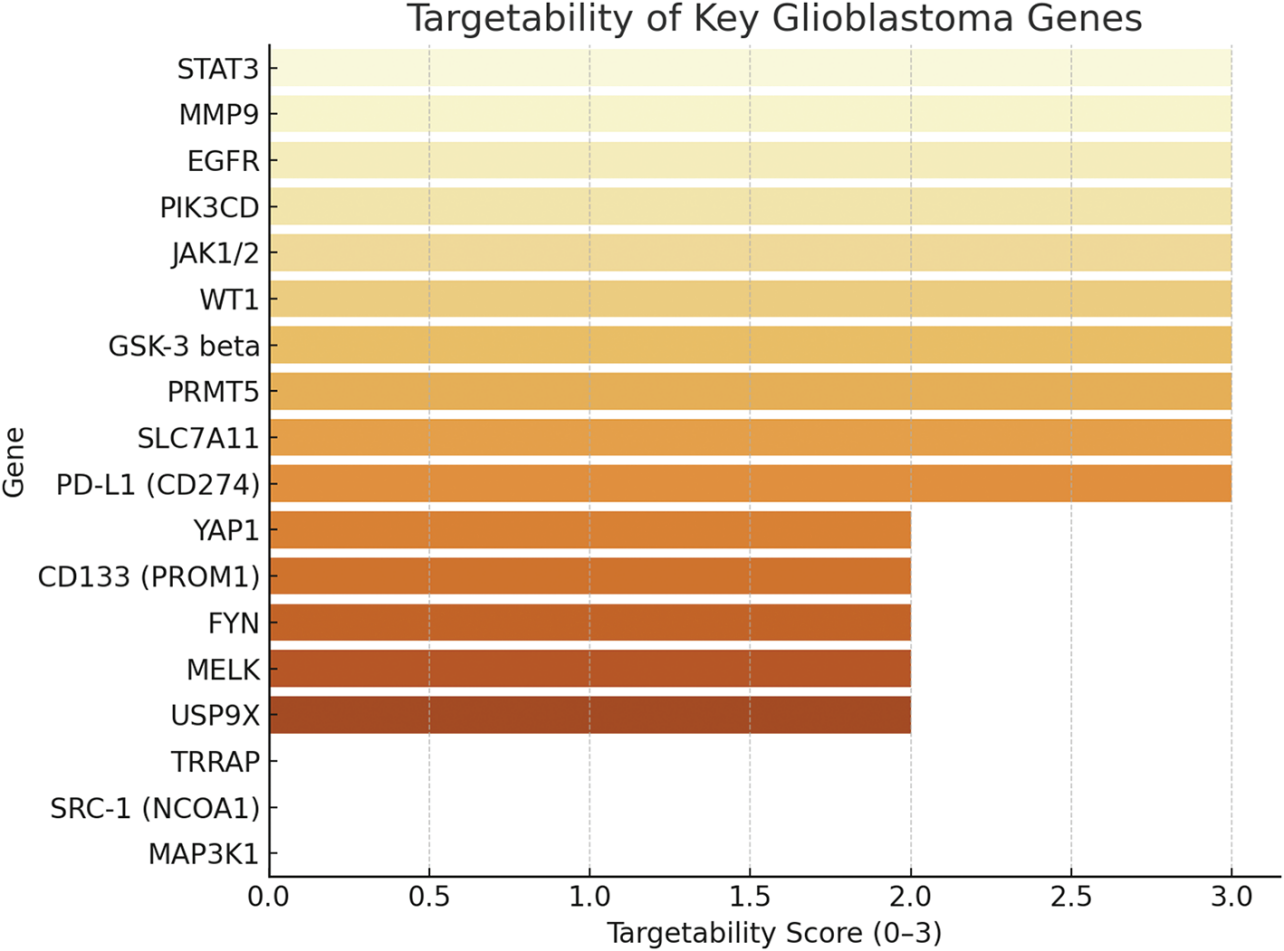
This figure shows the targetability scores of key glioblastoma genes, ranked from most to least targetable. The score (0–3) reflects: +1: drug availability, +1: inclusion in clinical trials +1: confirmed or partial blood-brain barrier (BBB) penetration. Genes like STAT3, MMP9, EGFR, PIK3CD, JAK1/2, and WT1 achieved the highest score [3], indicating strong translational potential with drugs, trials, and brain penetrance. And genes like MAP3K1, SRC-1, and TRRAP scored 0 or 1, representing underexplored targets

Targetability assessment combined data on drug availability, clinical trial inclusion, and blood-brain barrier (BBB) penetration (Fig. 6). Targetability scores were assigned based on the presence of: (i) available pharmacological inhibitors or biologics (+1), (ii) inclusion in active or completed cancer clinical trials (+1), and (iii) evidence of blood–brain barrier penetration of candidate drugs in preclinical or clinical studies (+1). Scores ranged from 0 to 3, with higher values reflecting greater translational feasibility. *STAT3*, *PD-L1*, *PRMT5*, *SLC7A11*, and *JAK1/2* emerged as high-priority targets with strong translational potential, supported by existing inhibitors and advanced clinical development. In contrast, highly aggressive genes like *AGR2*, *NCAN*, and *HAS2* lack effective targeted therapies, highlighting critical gaps in the therapeutic landscape. Mapping aggression against targetability revealed alignment between high aggression and strong targetability for some genes, but also identified poorly targetable aggressive drivers, emphasising the need for novel therapeutic development.

In total, 63.4% of the identified genes were predominantly upregulated in GBM compared to non-tumour brain, while 28.6% were downregulated and 8.0% showed mixed or context-specific patterns. Approximately 52% of genes demonstrated a prognostic association with reduced overall survival, whereas 21% correlated with improved prognosis; the remainder had inconsistent associations across studies. These data are summarised in Fig. 2, which provides an overview of the prognostic distribution of all genes included in this review.

## 4 Discussion

This systematic review provides an integrative overview of gene expression dysregulation in glioblastoma across 200 studies. While transcriptomic analyses have revealed key insights into GBM pathophysiology, mRNA levels do not always reflect protein abundance [18]. Gene expression refers to the transcription of DNA into RNA, whereas protein expression reflects the final functional output of cellular signalling [19]. This discordance is clinically significant: genes like *EGFR* and *PDGFRA* may be highly transcribed but show limited protein expression due to miRNA repression, translational inefficiency, or post-translational degradation [18,20]. These findings emphasise the necessity of integrating transcriptomic and proteomic data to enhance the reliability of biomarkers and therapeutic targets. For instance, Liu et al. demonstrated that mRNA-protein correlation is modest across tissues, reinforcing the need to validate transcript-based markers at the protein level [18,21]. In GBM, where both transcriptomic signatures and treatment responses are heterogeneous, dual-layer molecular profiling offers a more precise understanding of tumour behaviour. For immune modulators, this integration is particularly relevant: PD-L1 mRNA overexpression in GBM is often mirrored by elevated protein levels detected via IHC, whereas major histocompatibility complex (MHC)-I transcripts may be variably downregulated without consistent loss of surface protein [17,21,22]. Likewise, TAM-associated STAT3 and TGFB1 show strong concordance between transcriptomic enrichment and proteomic activation in the GBM microenvironment, reinforcing their biological relevance [23].

### 4.1 Stemness and Therapy Resistance in Glioblastoma

Glioblastoma (GBM) is characterised by extensive cellular heterogeneity, partly driven by stem-like subpopulations that resist therapy and fuel recurrence. Anterior Gradient 2 (*AGR2*) encodes a protein disulphide isomerase that, under physiological conditions, supports protein folding within the endoplasmic reticulum. In glioblastoma (GBM), *AGR2* is significantly overexpressed, disrupting cellular homeostasis and fostering malignant transformation. Its elevated expression in GBM tissues and cancer stem cell populations correlates with enhanced proliferation, invasion, and therapeutic resistance. *AGR2*’s co-expression with stemness markers such as Nestin and Vimentin highlights its role in maintaining glioma stem cell survival and contributing to recurrence, positioning AGR2 as a potential diagnostic and therapeutic target [24,25].

*SOX2* encodes a key transcription factor maintaining stem cell pluripotency [26]. In GBM, its dynamic, oscillatory expression supports transitions between proliferative and quiescent states, enabling heterogeneity and resistance [27,28]. *SOX2* features prominently in gene panels for GBM classification due to its central role in tumour aggressiveness [29,30].

*CD133 (PROM1)* marks a subpopulation of glioblastoma stem cells with potent tumour-initiating and chemoresistant properties [31]. These cells co-express genes including *BCRP1* (*ABCG2*), which facilitates drug efflux and chemotherapy resistance [32]; Nestin and Musashi-1 (Msi1), which support proliferation and stemness [33,34]; and MELK, which promotes cell cycle progression [35]. Together, these factors drive glioblastoma’s resilience and recurrence. *SOX10*, silenced by promoter methylation in GBM, further reflects disruption of neural differentiation programs and correlates with poor survival [36]. *ELAVL2*, an RNA-binding protein stabilising neuronal mRNAs, is downregulated in GBM, facilitating mesenchymal transition, invasion, and poor prognosis [37–39].

*OXCT1-AS1*, a long non-coding RNA, is markedly upregulated in GBM and contributes to oncogenesis by sponging miR-195 and derepressing *CDC25A*, enhancing proliferation and invasiveness [40]. Its post-transcriptional regulatory role makes *OXCT1*-*AS1* a compelling molecular target for therapeutic strategies [41,42]. *DCX, OLIG2*, and *NES* reflect the neural lineage features co-opted by GBM. *DCX*, involved in neuronal migration, is expressed in only rare tumour cells and appears unrelated to invasion [43,44]. *OLIG2* is enriched at tumour margins, supporting stemness and invasion [44]. *NES* is broadly expressed in undifferentiated GBM cells, facilitating proliferation and aggressiveness [44,45]. *ZDHHC18* and *ZDHHC23*, encoding palmitoyl acyltransferases, are differentially enriched in mesenchymal and proneural GBM subtypes, respectively. Both enzymes sustain glioma stem cell plasticity and survival, modulating *BMI1* stability and contributing to adaptability and therapy resistance [46–48].

### 4.2 Immune Evasion and Modulation

*CD274 (PD-L1)* facilitates immune escape in glioblastoma by inhibiting T-cell activity. Its variable expression correlates with poor prognosis and therapy resistance [49,50]. *PD-L1’s* dynamic regulation across the cell cycle suggests that immune evasion in GBM is tightly linked to proliferative cues, influencing immunotherapy responsiveness [51]. *MHC-I* genes (*HLA-A, HLA-B, HLA-C*) and *B2M* are frequently downregulated in GBM, impairing antigen presentation and promoting immune evasion [49,52]. *B2M* is overexpressed in gliomas, correlates with tumor grade, immune infiltration, and mesenchymal subtype; lower expression predicts longer survival, highlighting *B2M* as a prognostic biomarker and potential therapeutic target [53,54]. Loss of *TAP1* and *TAP2* further reduces tumour immunogenicity by limiting peptide loading onto *MHC*-I [55–57]. *CD40*, which is taken over by tumour cells, further facilitates invasion in chemoresistant GBM [58–60]. Collectively, these alterations contribute to poor prognosis while decoupling immune escape from standard therapy responses [16,61].

### 4.3 Immune Checkpoint Blockade in Glioblastoma: Current Clinical Experience

While GBM tumours frequently exploit immune evasion mechanisms such as PD-L1 overexpression, MHC-I downregulation, and impaired antigen presentation via TAP1/TAP2 suppression, translation of these findings into effective immune checkpoint inhibitor (ICI) therapies has proven challenging [62]. Trials targeting PD-1/PD-L1 (e.g., CheckMate 143) and CTLA-4 have demonstrated limited clinical benefit in unselected GBM populations, largely due to the profoundly immunosuppressive tumor microenvironment [63]. Ongoing studies (e.g., NCT02667587, NCT02337491) are testing combination regimens integrating ICIs with radiotherapy, personalized vaccines, or metabolic modulators to enhance immune activation [64,65]. These efforts highlight the need for precise patient selection and biomarker integration to improve therapeutic outcomes.

### 4.4 Transcriptomic Correlates of the GBM Immune Microenvironment

The glioblastoma immune milieu is dominated by tumour-associated macrophages (TAMs), most of which adopt an M2-like phenotype that supports tumour growth and suppresses antitumor immunity [66]. Transcriptomic profiling reveals consistent upregulation of *STAT3*, *IL10*, and *TGFB1*, which drive M2 polarisation, inhibit cytotoxic T-cell function, and foster immune tolerance [67]. Tumour-infiltrating lymphocytes (TILs) exhibit elevated expression of exhaustion markers such as *PDCD1*, *LAG3*, and *HAVCR2*, accompanied by reduced effector cytokines, reflecting chronic antigen exposure [68]. High-resolution single-cell RNA sequencing has mapped these immune cell states in GBM, showing convergence between immune transcriptomes and tumor-intrinsic expression programs. This interplay underscores the role of immune–tumor crosstalk in shaping disease progression and resistance to immunotherapy [69].

### 4.5 Translational Immunotherapy Strategies and Patient Stratification

Building on these mechanistic insights, several translational strategies aim to circumvent GBM’s immune resistance. Oncolytic virotherapies such as DNX-2401 can induce immunogenic cell death and enhance antigen presentation. Dendritic cell vaccines, exemplified by ICT-107, are designed to amplify tumor-specific T-cell responses [70]. Metabolic interventions, including inhibition of IDO1-mediated tryptophan catabolism or targeting lactate accumulation, may reprogram the microenvironment to favor immune activation. Patient stratification based on PD-L1 status, tumor mutational burden, or immune-related transcriptomic signatures could refine trial design and improve response prediction [71,72]. Integrating such biomarker-driven approaches with adaptive immunotherapy regimens holds promise for translating immune modulation into durable survival benefits for GBM patients.

Notably, several GBM-associated genes display discordance between mRNA and protein abundance, underscoring the need for multi-omics validation. For instance, *EGFR* often shows high mRNA expression without proportional protein overexpression due to post-transcriptional regulation or receptor degradation [6]. Similarly, *PDGFRA* transcriptional upregulation may not translate into increased protein levels, potentially reflecting microRNA-mediated suppression [73]. In contrast, *VEGFA* mRNA and protein levels can diverge in hypoxic microenvironments, where translational control mechanisms predominate [74]. These examples highlight the limitations of relying solely on transcriptomic data and support integrating proteomic or immunohistochemical evidence when prioritising therapeutic targets.

### 4.6 Extracellular Matrix (ECM) Remodeling and Angiogenesis

Invasion and angiogenesis in GBM are mediated by coordinated extracellular matrix (ECM) remodeling and pro-angiogenic signaling. VEGFA is a key driver of angiogenesis in GBM [75]. Its overexpression supports abnormal vasculature, tumor growth, and hypoxia adaptation [75,76]. Interestingly, *VEGFA* levels predict bevacizumab response, marking it as both a pathogenic driver and therapeutic marker [77].

*Ephrin-B2* promotes proliferation, migration, and invasion, with high expression linked to poor survival. Furthermore, high *ephrin-B2* expression serves as a strong predictor of shorter survival in glioma patients, underscoring its potential as a prognostic biomarker [78]. Targeting ephrin-B2 signaling pathways may offer therapeutic strategies to mitigate GBM aggressiveness [79].

*MMP2, MMP9*, and *MMP14* degrade ECM components, facilitating invasion and angiogenesis [80,81]. Despite co-expression of their inhibitors (*TIMP1, TIMP2*), the proteolytic balance in GBM favours invasion [82–84]. *COL1A2, IGFBP3, NGFR*, and *WIF1. COL1A2* encodes the alpha-2 chain of type I collagen, a key component of the extracellular matrix; its overexpression may facilitate tumor invasion and has been linked to reduced overall survival in GBM patients [85,86]. *IGFBP3* regulates insulin-like growth factors, influencing cell growth and apoptosis; elevated levels in GBM correlate with poorer survival outcomes [87]. NGFR, involved in neuronal survival and apoptosis, shows increased expression in GBM, which is associated with decreased patient survival [88]. Conversely, *WIF1* acts as an antagonist of the Wnt signaling pathway, and its reduced expression in GBM is linked to shorter survival, suggesting a tumor suppressor role [86,89].

*COL5A1, COL5A3, COL27A1* [82,85,86,90], Laminins (*LAMA1, LAMB1, LAMB2, LAMC1*) [82,91], Fibulins (*FBLN1, FBLN2*) [82,92], Integrins (*ITGA3, ITGA5, ITGB1*) [82,93], and *HAS1/2* all contribute to matrix remodeling, angiogenesis, and therapy resistance [82,94]. High expression of these genes correlates with reduced survival and supports GBM’s mesenchymal phenotype. *TGFB1* and *WNT9B* drive angiogenesis and stem-like endothelial characteristics, supporting vascular mimicry and resistance [82,95,96]. *PDCD10* loss enhances GBM progression by upregulating EPHB4 kinase activity, promoting proliferation, migration, and angiogenesis [97–99]. *NCAN* uniquely associates with better survival, possibly via anti-angiogenic effects [82,100].

### 4.7 Metabolic Reprogramming and Redox Balance

*SLC7A11* encodes a cystine/glutamate antiporter supporting glutathione synthesis and oxidative stress resistance in GBM [101]. Its overexpression promotes redox balance but suppresses mismatch repair, increasing genomic instability [102]. *HIF-1*α facilitates adaptation to hypoxia, driving VEGF-mediated angiogenesis and metabolic reprogramming [103,104]. Cooperation between HIF-1α-positive and -negative cells enhances heterogeneity and tumor growth [105]. Inhibition of *NRF2* enhances response to temozolomide (TMZ) and radiotherapy, highlighting it as a metabolic checkpoint in glioblastoma treatment [106]. Kurdi et al. (2023) reported significant dysregulation of *NFE2L2* in IDH-mutant astrocytomas, suggesting that NRF2 may interact with metabolic reprogramming driven by *IDH1* mutations [107]. This crosstalk contributes to tumor adaptability and survival, making *NFE2L2* a potential therapeutic target [107].

### 4.8 Epigenetic and Post-Transcriptional Dysregulation

Epigenetic and post-transcriptional alterations play central roles in GBM progression. Genes like *ZNF217, FABP7*, and *GSTM5* show inverse methylation-expression patterns, underscoring the contribution of methylation dynamics to GBM heterogeneity [108]. Mu et al. (2025) found that *TRRAP* is stabilised by USP9X-mediated deubiquitination, enhancing its oncogenic activity. Elevated *TRRAP* levels lead to increased expression of genes that drive tumour progression. Its role in glioblastoma extends beyond transcription to influencing immune interactions, marking *TRRAP* as a novel effector in GBM pathogenesis [109]. *NDRG2* is a tumour suppressor gene that plays a role in cell differentiation, stress response, and the inhibition of cell proliferation. In grade 4 astrocytomas, *NDRG2* expression is frequently downregulated, contributing to tumour progression, increased invasiveness, and reduced apoptosis [110]. Kurdi et al. (2023) reported that low *NDRG2* expression, particularly in combination with *IDH1* mutation, is associated with more aggressive tumour features. This synergistic dysregulation appears to weaken tumour-suppressive mechanisms, highlighting *NDRG2* as a potential prognostic biomarker and therapeutic target in high-grade gliomas [107].

### 4.9 Chemoresistance and Cytoskeletal Dynamics

*LAPTM5* upregulation in TMZ-resistant cells supports oncogenic trafficking [59,111], working with *CD40* to promote invasion and chemoresistance [46–48]. *TUBB6* and *FYN*, co-regulated with *LAPTM5*, enhance cytoskeletal reorganisation and invasion [59,112,113]. *LINGO1* [25,114,115] and *C7orf31* are linked to poor survival, with roles in adhesion signaling and genomic stability still under investigation [114,116] Other genes, such as *ZNF217, FABP7*, and *GSTM5*, exhibit inverse methylation-expression patterns, indicating that epigenetic silencing of these oncogenic or metabolic regulators may also shape glioma behaviour [108] Similarly, *TUBB6*, *DTX1*, and integrin subunits (*ITGA3, ITGA5, ITGB1*) support cytoskeletal remodeling and motility through actin dynamics and focal adhesion pathways [117,118].

### 4.10 Key Signalling Pathways and Transcriptional Regulation

IL6/STAT3 signalling is hyperactivated in GBM, promoting proliferation, immune evasion, and therapy resistance [119–122]. Loss of *SOCS3* amplifies STAT3 activity [123]. *NOTCH1, HES1, DTX1*, and *TRIM8* sustain stemness and invasion through dysregulated Notch signalling and STAT3 hyperactivation [124,125]. *PIK3CD, PAK3*, and *PLEK2* drive actin remodelling, migration, and invasion via PI3K signalling [126–128]. *MAP3K1* and *TRIB2* cooperate to promote temozolomide resistance and survival signalling [129–131]. *PRMT5* epigenetically regulates gene expression and RNA splicing, supporting GBM cell survival and resistance [132,133]. *USP9X* stabilises TRRAP, enhancing chromatin remodelling and immune evasion through M2 macrophage polarisation [109,134]. *TMBIM1* modulates p38 *MAPK* signalling to promote survival and inhibit apoptosis [135,136]. *SRC*-*1* (*NCOA1*) rewires corticosteroid responses toward tumour promotion under steroid treatment [137]. *ZEB2* drives epithelial-to-mesenchymal transition, invasion, and apoptosis resistance [138]. *YAP1* enhances growth, stemness, and resistance [117,139]. *GSK*-*3β* shows duality: it promotes cholera toxin-induced differentiation when active [140], but its inhibition reduces proliferation and invasion, reflecting context-dependent therapeutic relevance [141–143]. WT1 displays dual roles in GBM, correlating with poor prognosis in IDH-wildtype tumours but associating with longer recurrence-free survival in IDH-mutants receiving chemotherapy [144,145]

## 5 Therapeutic Targets and Translational Opportunities

The integrated analysis of glioblastoma gene expression reveals several promising therapeutic targets with translational potential. *VEGFA*, as a central angiogenic driver, remains a key target, with anti-VEGF therapies like bevacizumab showing benefit in subsets of patients with high VEGFA expression [75]. Similarly, *PD-L1 (CD274)* represents a well-established immune checkpoint target; its dynamic regulation suggests that optimizing the timing and combination of immune therapies could enhance efficacy [49]. Disruption of ECM remodeling enzymes, notably *MMP14, MMP2*, and *MMP9*, alongside their paradoxically upregulated inhibitors *TIMP1* and *TIMP2*, offers avenues to curb invasion and angiogenesis [146,147].

Epigenetic regulators like *PRMT5* and *USP9X* present attractive targets for impairing GBM cell survival and immune evasion [133]. Similarly, blocking *STAT3* or restoring *SH3GL2* expression may limit invasiveness [119,148]. The Notch pathway (via *NOTCH1*, *HES1*, *DTX1*) and its interaction with *TRIM8* and STAT3 signaling represent another axis for potential intervention, particularly in glioma stem-like cells. The study positions *TSC22D1* as a candidate tumor suppressor whose inactivation may play a critical role in glioblastoma pathogenesis and progression [149]. Novel candidates like *OXCT1*-*AS1*, *WNT9B*, and *SRC*-*1* open fresh avenues for targeting post-transcriptional regulation, angiogenesis, and steroid-driven tumor adaptation.

Importantly, *WT1* and *ZEB2* emerge as biomarkers for patient stratification and as potential direct targets, given their links to prognosis and therapy resistance [138,139]. Together, these insights underline the urgent need for biomarker-driven, combination therapies that disrupt the molecular circuits sustaining glioblastoma progression. Isoform-specific inhibitors, metabolic modulators, and agents targeting the tumour microenvironment (e.g., ECM, immune milieu) represent key translational opportunities to enhance GBM treatment outcomes.

Our integrative synthesis advances the field by providing a functional and subtype-oriented map of glioblastoma gene expression. Unlike previous reviews that examined individual pathways or limited gene sets, this work compiles 112 GBM-relevant genes. It categorises them into stemness, immune modulation, ECM/invasion, metabolic regulation, and epigenetic control. We further mapped these genes to the four established transcriptional subtypes (classical, mesenchymal, proneural, and neural), visualised in a newly constructed subtype heatmap (Fig. 5). This visualisation reveals clear patterns of subtype enrichment, for example, the clustering of OLIG2, SOX2, and Nestin within the proneural group, and the predominance of MMP2, MMP9, and COL5A1 in the mesenchymal subtype, providing a basis for tailoring therapeutic strategies to molecularly defined patient groups.

By integrating functional categorisation with subtype distribution, our analysis links gene expression patterns directly to glioblastoma’s biological hallmarks and potential therapeutic vulnerabilities. This approach also incorporates awareness of transcript–protein discrepancies, emphasising that subtype-associated targets should undergo proteomic and immunohistochemical validation before clinical translation. Such a framework enables the identification of context-specific intervention points, whether through targeting stemness-associated transcription factors in proneural GBM or ECM-remodelling enzymes in mesenchymal GBM. It supports the rational design of biomarker-driven, combination therapies that can be adapted to the molecular context of each patient.

## 6 Limitations

Our review synthesised transcriptomic data from multiple publicly available repositories and literature sources, covering 112 glioblastoma-relevant genes. Quantitative descriptive analyses were limited to the 85 genes with complete expression and functional annotations across datasets. Several significant constraints should be noted. First, while a formal meta-analysis could provide stronger statistical inference, substantial heterogeneity in patient cohorts, sequencing platforms, clinical endpoints, and reporting formats precluded reliable pooling of hazard ratios without introducing bias. We therefore adopted a qualitative synthesis supplemented with descriptive statistics to preserve methodological rigour. Second, the functional characterisation of many genes, particularly long non-coding RNAs and less-studied transcriptional regulators, remains incomplete, underscoring the need for additional mechanistic and preclinical validation. Third, discrepancies between mRNA abundance and protein levels, as seen for genes such as EGFR, PDGFRA, and VEGFA, highlight the importance of incorporating proteomic and immunohistochemical data in future studies. Fourth, glioblastoma’s pronounced inter- and intra-tumoral heterogeneity complicates the generalisation of transcriptomic signatures across all patient populations. Finally, although we have provided subtype-specific visualisations, further integrative multi-omics profiling, functional assays, and clinically annotated datasets will be essential to translate these molecular insights into robust, subtype-tailored therapeutic strategies.

## 7 Conclusion

This comprehensive analysis highlights the intricate molecular landscape of glioblastoma, characterised by dysregulation of stemness pathways, immune evasion mechanisms, extracellular matrix remodelling, metabolic reprogramming, and key signalling networks. Genes such as VEGFA, PD-L1, PIK3CD, PRMT5, and STAT3 emerge as central nodes driving glioblastoma progression and therapeutic resistance. Novel candidates, including OXCT1-AS1, SRC-1, and WNT9B, represent emerging targets with potential to complement existing therapies. These findings underscore the critical need for biomarker-driven, combinatorial treatment strategies to improve glioblastoma outcomes.

## Supplementary Materials



## Data Availability

Not applicable.

## Supplementary Materials

The supplementary material is available online at https://www.techscience.com/doi/10.32604/or.2025.070031/s1.

## Abbreviations

BBB: Blood-brain barrier
CGGA: Chinese Glioma Genome Atlas
CNS: Central nervous system
ECM: Extracellular matrix
EGFR: Epidermal growth factor receptor
EMT: Epithelial-mesenchymal transition
FGFR3: Fibroblast growth factor receptor 3
GBM: Glioblastoma
GSC: Glioma stem cell
HIF-1α: Hypoxia-inducible factor 1 alpha
IDH: Isocitrate dehydrogenase
lncRNA: Long non-coding RNA
MET: Mesenchymal-epithelial transition
MHC: Major histocompatibility complex
MMP: Matrix metalloproteinase
mRNA: Messenger RNA
NGS: Next-generation sequencing
NRF2: Nuclear factor erythroid 2-related factor 2
PDGFRA: Platelet-derived growth factor receptor alpha
PI3K: Phosphoinositide 3-kinase
PRMT5: Protein arginine methyltransferase 5
ROS: Reactive oxygen species
RTK: Receptor tyrosine kinase
scRNA-seq: Single-cell RNA sequencing
SLC7A11: Solute carrier family 7 member 11
SOCS3: Suppressor of cytokine signalling 3
STAT3: Signal transducer and activator of transcription 3
TAM: Tumour-associated macrophage
TAP: Transporter associated with antigen processing
TCGA: The Cancer Genome Atlas
TERT: Telomerase reverse transcriptase
TIL: Tumour-infiltrating lymphocyte
TIMP: Tissue inhibitor of metalloproteinases
TP53: Tumor protein p53
TRIM8: Tripartite motif containing 8
USP9X: Ubiquitin-specific peptidase 9 X-linked
VEGFA: Vascular endothelial growth factor A
WHO: World Health Organisation

## Appendix A

**Table A1:** Stemness-related genes in GBM

Gene	Expression in GBM	Functional category	Prognostic relevance
CD133 (PROM1)	Upregulated	Stemness	Associated with poor survival
Nestin (NES)	Upregulated	Stemness	Linked to tumor initiation
Musashi-1 (MSI1)	Upregulated	Stemness	Correlates with aggressive phenotype
SOX2	Expression reduced when methylated	Stemness	Lower expression linked to better outcome
OLIG2	Upregulated	Stemness	Proneural subtype marker
WT1	Upregulated	Stemness	Stem-like cell expression

**Table A2:** Proliferation/Survival-related genes in GBM

Gene	Expression in GBM	Functional category	Prognostic relevance
MELK	Upregulated	Proliferation/Survival	Linked to tumor growth
ZDHHC18	Upregulated	Proliferation/Survival	Correlates with GBM aggressiveness
ZDHHC23	Upregulated	Proliferation/Survival	Associated with stemness regulation
EphrinB2	Upregulated	Proliferation/Survival	Angiogenesis and migration
Dll4	Upregulated	Proliferation/Survival	Enhances proliferation
Jag1	Upregulated	Proliferation/Survival	Notch signaling activation
HES1	Upregulated	Proliferation/Survival	Poor prognosis
DTX1	Upregulated	Proliferation/Survival	Poor survival
YAP1	Upregulated in MGMT-methylated GBM	Proliferation/Survival	Better survival in MGMT-methylated GBM
STAT3	Upregulated	Proliferation/Survival	Mixed evidence (context dependent)
JAK1/2	Overexpressed	Proliferation/Survival	Correlates with tumor aggressiveness
SOCS3	Overexpressed	Proliferation/Survival	High-grade association
LAPTM5	Highly upregulated	Proliferation/Survival	Tumor grade and prognosis correlation
CD40	Strongly expressed	Proliferation/Survival	Aggressive phenotype
FYN	Upregulated	Proliferation/Survival	Enhances proliferation
PDCD10	Upregulated	Proliferation/Survival	Tumor angiogenesis
EPHB4	Upregulated	Proliferation/Survival	Endothelial remodeling
PRMT5	Upregulated	Proliferation/Survival	Growth in PDCD10-deficient tumors
SRC-1 (NCOA1)	Hyper activated	Proliferation/Survival	Invasive phenotype in SH3GL2-deficient GBM
TMBIM1	Upregulated via STAT3	Proliferation/Survival	Survival mechanism activation
TRRAP	Highly upregulated	Proliferation/Survival	Oncogenic co-activator
TRIB2	Variable; linked to low MMR gene expression	Proliferation/Survival	Unfavorable with low mismatch repair
TRIM8	Time-dependent fluctuations	Proliferation/Survival	Fluctuates with disease stage
USP9X	Overexpressed in 14%, down in 86%	Proliferation/Survival	Mixed survival correlation
TSC-22	Highly expressed	Proliferation/Survival	Aggressive GBM phenotype
ZEB2	Upregulated	Proliferation/Survival	Enhances GBM progression

**Table A3:** ECM/Invasion-related genes in GBM

Gene	Expression in GBM	Functional category	Prognostic relevance
COL1A2	Frequently mutated/deleted	ECM/Invasion	Tumor invasiveness marker
COL5A1	Overexpressed	ECM/Invasion	Poor prognosis
COL5A3	Overexpressed	ECM/Invasion	Poor prognosis
COL27A1	Downregulated in aggressive GBM	ECM/Invasion	Linked to IDH mutation effects
LAMA1	Highly expressed	ECM/Invasion	Invasive subtype marker
LAMB1	Highly expressed	ECM/Invasion	Invasive subtype marker
LAMB2	Highly expressed	ECM/Invasion	Invasive subtype marker
LAMC1	Highly expressed	ECM/Invasion	Invasive subtype marker
FBLN1	Upregulated	ECM/Invasion	Up in GBM ECs
FBLN2	Upregulated	ECM/Invasion	Endothelial activation
ITGA3	Upregulated	ECM/Invasion	Linked to better survival
ITGA5	Upregulated	ECM/Invasion	Linked to better survival
ITGB1	Upregulated	ECM/Invasion	Decreased in recurrent GBM
MMP9	Upregulated	ECM/Invasion	Associated with poor survival
MMP2	Upregulated	ECM/Invasion	Upregulated with proliferation
TIMP1	Upregulated	ECM/Invasion	Poor prognosis in ECs
TIMP2	Upregulated	ECM/Invasion	Poor prognosis in ECs
TGFB1	Upregulated	ECM/Invasion	Poor prognosis and immunosuppression
TUBB6	Upregulated	ECM/Invasion	Linked to bevacizumab response
WNT9B	Upregulated	ECM/Invasion	Up in both ECs and tumor cells
MT1-MMP (MMP14)	Reduced in TMZ-resistant GBM	ECM/Invasion	Down in recurrent/therapy-resistant GBM
NCAN	Upregulated in aggressive GBM	ECM/Invasion	Aggressive GBM phenotype
HAS1	Upregulated	ECM/Invasion	Mesenchymal GBM feature
HAS2	Upregulated	ECM/Invasion	Mesenchymal GBM feature

**Table A4:** Immune modulation-related genes in GBM

Gene	Expression in GBM	Functional category	Prognostic relevance
IL-6	Upregulated	Immune modulation	Associated with poor prognosis
STAT3	Downregulated	Immune modulation	Downregulation linked to poor survival
JAK1/2	Overexpressed	Immune modulation	Correlates with tumor immune signaling
SOCS3	Overexpressed	Immune modulation	Correlates with high-grade GBM
CD40	Strongly expressed	Immune modulation	Linked to aggressive immune phenotype
LAPTM5	Highly upregulated	Immune modulation	Linked to prognosis and grade
MHC-I	High in LAPTM5-low GBM	Immune modulation	Poor prognosis indicator
TAP1	Low in long-term survivors	Immune modulation	Better outcome in long-term survivors
TAP2	High in short-term survivors	Immune modulation	Linked to poor outcome
CD274 (PD-L1)	Upregulated in LAPTM5-related clusters	Immune modulation	Tumor immune evasion
B2M	Highly expressed in long-term survivors	Immune modulation	Survival in long-term patients
WIF1	Highly expressed in GBM stem-like cells	Immune modulation	Stemness and immune role
NGFR	Homozygous deletions common	Immune modulation	Associated with therapy resistance
LINGO1	Upregulated	Immune modulation	Immune regulation and GBM signaling

**Table A5:** Metabolic regulation-related genes in GBM

Gene	Expression in GBM	Functional category	Prognostic relevance
OXCT1-AS1	Upregulated; linked to poor prognosis	Metabolic regulation	Associated with poor outcomes
GSK-3 beta (GSK3B)	Upregulated; linked to shorter OS	Metabolic regulation	Correlates with poor survival
NRF2 (NFE2L2)	Increased expression; higher tumor grade	Metabolic regulation	High-grade GBM marker
PIK3CD	Upregulated in 70% of tumors	Metabolic regulation	Linked to IDH mutation
PAK3	Mutations in 21/34 tumors	Metabolic regulation	Mutational status linked to better prognosis
MAP3K1	Inversely correlated with SLC7A11	Metabolic regulation	Regulates oxidative stress
SLC7A11	Peaks with PD-L1 during active cycle	Metabolic regulation	Tumor nutrient uptake
TRIB2	High expression linked to low MMR expression	Metabolic regulation	Low mismatch repair expression
HAS1	Upregulated in GBM and ECs	Metabolic regulation	Mesenchymal subtype marker
HAS2	Upregulated in GBM and mesenchymal subtype	Metabolic regulation	Mesenchymal and immune phenotype
WNT9B	Upregulated in ECs and tumor cells	Metabolic regulation	EC and tumor cell regulation

**Table A6:** Epigenetic control-related genes in GBM

Gene	Expression in GBM	Functional category	Prognostic relevance
SOX2	Expression reduced when methylated	Epigenetic control	Methylation linked to lower expression and poorer outcomes
SH3GL2	Overexpressed; co-expressed with PDGFRA	Epigenetic control	Enhances growth signaling pathways
PRMT5	Upregulated in PDCD10-deficient tumors	Epigenetic control	Linked to poor prognosis via epigenetic silencing
USP9X	Overexpressed in 14%, down in 86%	Epigenetic control	Tumor suppressor and oncogene dual role
TRIM8	Time-dependent fluctuations	Epigenetic control	Dynamic expression may reflect tumor stage
TRRAP	Highly upregulated	Epigenetic control	Chromatin regulator in aggressive tumors

**Table A7:** Aggression score stratification of key glioblastoma genes

Gene symbol	Aggression score	Expression status	Prognostic association	Group
AGR2	1.0	Upregulated	Poor prognosis	Top 20
NCAN	1.0	Upregulated	Poor prognosis	Top 20
TIMP1/TIMP2	1.0	Upregulated	Poor prognosis	Top 20
MMP9	1.0	Upregulated	Reduced survival	Top 20
HAS1/HAS2	1.0	Upregulated	Poor prognosis (mesenchymal)	Top 20
FBLN1/FBLN2	1.0	Upregulated	Pro-tumorigenic (ECs)	Top 20
LAMA1–LAMB1–LAMB2–LAMC1	1.0	Highly expressed	Invasive phenotype	Top 20
COL5A1/COL5A3	1.0	Overexpressed	Poor prognosis	Top 20
COL27A1	1.0	Downregulated	Aggressive GBM (IDH-linked)	Top 20
C7orf31	1.0	Decreased	Poor prognosis	Top 20
NDRG2	1.0	Downregulated	Poor prognosis	Top 20
NRF2	1.0	Increased	Higher grade glioma	Top 20
DTX1	1.0	Elevated	Poor survival	Top 20
HES1	1.0	Upregulated	Poor prognosis	Top 20
OXCT1-AS1	1.0	Upregulated	Negative prognostic marker	Top 20
MT1-MMP	1.0	Reduced	TMZ-resistance	Top 20
TGFB1	1.0	Upregulated	Pro-tumorigenic	Top 20
WNT9B	1.0	Upregulated	Angiogenic activation	Top 20
FYN	1.0	Upregulated	Invasion/metastasis	Top 20
PDCD10	1.0	Upregulated	Tumor progression	Top 20
STAT3	−1.0	Downregulated	Better survival (contextual)	Bottom 20
LAPTM5	−1.0	Upregulated	Improved outcome (paradoxical)	Bottom 20
CD40	−1.0	Strongly expressed	Linked to immune activation	Bottom 20
SH3GL2	−1.0	Overexpressed	Protective (with PDGFRA)	Bottom 20
USP9X	−1.0	Downregulated (86%)	Context-dependent	Bottom 20
IGFBP3	−1.0	Altered	Variable	Bottom 20
TAP1/TAP2	−1.0	Low in long-term survivors	Prognostic indicator	Bottom 20
PIK3CD	−1.0	Upregulated (70%)	Associated with IDH mutation	Bottom 20
PLEK2	−1.0	Inferred dysregulation	Poor outcome	Bottom 20
TUBB6	−1.0	Upregulated	Response to bevacizumab	Bottom 20
SOX2	−1.0	Methylation-sensitive	Improved survival when methylated	Bottom 20
TRIM8	−1.0	Fluctuating	Stress response	Bottom 20
B2M	−1.0	Highly expressed	Long-term survival	Bottom 20
ITGA3/ITGA5/ITGB1	−1.0	Upregulated	Improved survival (ITGB1 subset)	Bottom 20
YAP1	−1.0	Upregulated	Better survival (MGMT+)	Bottom 20
Notch1	−1.0	Elevated	Improved survival	Bottom 20
OLIG2	−1.0	Upregulated	Stem-like subtype	Bottom 20
PAK3	−1.0	Mutated in 21/34 tumors	Better prognosis	Bottom 20
WIF1	−1.0	Highly expressed	Stem-like GBM	Bottom 20
VEGFA	−1.0	Expressed in CD40+ GBM	Favorable in CD40+ subtype	Bottom 20
