# Supplementary material for: RNA Expression Signatures in Glioblastoma: A Systematic Review of Tumour Biology and Therapeutic Targets
Source: Oncol Res. 2025 Oct 22;33(11):3293–325. doi: 10.32604/or.2025.070031 (PMC12573197; doi:10.32604/or.2025.070031)
Supplement: Supplementary file 1 [file OncolRes-33-70031-s001.docx]

| **Section and Topic** | **Item No** | **Checklist item** | **Reported on Page Number/Line Number** | **Reported on Section/Paragraph** |
| --- | --- | --- | --- | --- |
| **TITLE** | | |  |  |
| Title | 1 | Identify the report as a systematic review. | 1/LINE 1 | TITLE |
| **ABSTRACT** | | |  |  |
| Abstract | 2 | See the PRISMA 2020 for Abstracts checklist. | Page 2, Lines 41–65 | (Abstract) |
| **INTRODUCTION** | | |  |  |
| Rationale | 3 | Describe the rationale for the review in the context of existing knowledge. | PAGE 4, LINE 126-135 | INTRODUCTION |
| Objectives | 4 | Provide an explicit statement of the objective(s) or question(s) the review addresses. | PAGE 4-5, LINE 132-134 | INTRODUCTION |
| **METHODS** | | |  |  |
| Eligibility criteria | 5 | Specify the inclusion and exclusion criteria for the review and how studies were grouped for the syntheses. | Page 5-6, Lines 159–164 | Methods, “Eligibility Criteria” |
| Information sources | 6 | Specify all databases, registers, websites, organisations, reference lists and other sources searched or consulted to identify studies. Specify the date when each source was last searched or consulted. | Page 6, Lines 170–179 | Methods, “Information Sources” |
| Search strategy | 7 | Present the full search strategies for all databases, registers and websites, including any filters and limits used. | Page 7, Lines 16–35 | Methods, “Search Strategy” |
| Selection process | 8 | Specify the methods used to decide whether a study met the inclusion criteria of the review, including how many reviewers screened each record and each report retrieved, whether they worked independently, and if applicable, details of automation tools used in the process. | Page 5, Lines 148–157 | Methods, “Selection Process” |
| Data collection process | 9 | Specify the methods used to collect data from reports, including how many reviewers collected data from each report, whether they worked independently, any processes for obtaining or confirming data from study investigators, and if applicable, details of automation tools used in the process. | Page 6.-7 Lines 181–129 | Methods, “Data Extraction, Curation and Scoring” |
| Data items | 10a | List and define all outcomes for which data were sought. Specify whether all results that were compatible with each outcome domain in each study were sought (e.g. for all measures, time points, analyses), and if not, the methods used to decide which results to collect. | Page 7–8, Lines 185–210 | Methods, “Data Extraction…” |
|  | 10b | List and define all other variables for which data were sought (e.g. participant and intervention characteristics, funding sources). Describe any assumptions made about any missing or unclear information. | Page 6-7, Lines 212–219 | Methods, “Data Extraction…” |
| Study risk of bias assessment | 11 | Specify the methods used to assess risk of bias in the included studies, including details of the tool(s) used, how many reviewers assessed each study and whether they worked independently, and if applicable, details of automation tools used in the process. | Not explicitly reported |  |
| Effect measures | 12 | Specify for each outcome the effect measure(s) (e.g. risk ratio, mean difference) used in the synthesis or presentation of results. | Not applicable – descriptive synthesis only |  |
| Synthesis methods | 13a | Describe the processes used to decide which studies were eligible for each synthesis (e.g. tabulating the study intervention characteristics and comparing against the planned groups for each synthesis (item #5)). |  | Data Extraction, Curation and Scoring |
|  | 13b | Describe any methods required to prepare the data for presentation or synthesis, such as handling of missing summary statistics, or data conversions. | PAGE 7 | *Methods*, “Data Validation |
|  | 13c | Describe any methods used to tabulate or visually display results of individual studies and syntheses. | PAGE 6 | *Methods*, end of “Data Extraction”  Results from each included study were compiled into structured gene annotation tables (Supplementary Tables 1-7), detailing expression trends, prognostic associations, and targetability scores. These data were visually summarised using multiple formats, including subtype-specific heatmaps, functional category bar charts, aggression–targetability dual-axis plots, and PRISMA diagrams, to facilitate interpretation of complex multi-source finding |
|  | 13d | Describe any methods used to synthesize results and provide a rationale for the choice(s). If meta-analysis was performed, describe the model(s), method(s) to identify the presence and extent of statistical heterogeneity, and software package(s) used. | Line 229-247, page 8 | *Methods*, “Data Validation |
|  | 13e | Describe any methods used to explore possible causes of heterogeneity among study results (e.g. subgroup analysis, meta-regression). | Page 8 | Heterogeneity among studies was qualitatively assessed based on differences in study design, patient cohort size, geographic origin, transcriptomic platform (e.g., RNA-seq vs. microarray), and clinical outcome definitions. This assessment informed the decision to adopt a descriptive synthesis rather than a pooled meta-analysis |
|  | 13f | Describe any sensitivity analyses conducted to assess robustness of the synthesized results. | Page 8 | Sensitivity analyses were not conducted due to the descriptive nature of the synthesis and substantial methodological heterogeneity across included studies |
| Reporting bias assessment | 14 | Describe any methods used to assess risk of bias due to missing results in a synthesis (arising from reporting biases). | Page 8 | Potential reporting bias was mitigated by including studies from both high-impact and specialty journals, conference proceedings where available, and by cross-validating results across independent datasets (TCGA, CGGA, GlioVis) to minimise the influence of selective reporting. |
| Certainty assessment | 15 | Describe any methods used to assess certainty (or confidence) in the body of evidence for an outcome. | Page 8 | Certainty in the body of evidence was qualitatively evaluated based on the degree of replication across independent datasets, consistency of prognostic associations, and the presence of experimental or clinical validation in the published literature |
| **RESULTS** | | |  |  |
| Study selection | 16a | Describe the results of the search and selection process, from the number of records identified in the search to the number of studies included in the review, ideally using a flow diagram. | Page 8, lines 251-269 | The search and selection process identified 410 unique records. After removing 90 duplicates, 320 titles and abstracts were screened, resulting in the exclusion of 121 records that did not meet eligibility criteria. A further 74 studies were excluded after full-text review due to insufficient gene-level data (n = 38), focus on non-GBM gliomas (n = 25), or non-primary research formats such as reviews or commentaries (n = 11). The final dataset included 125 studies (Fig. 1, PRISMA 2020 flow diagram) |
|  | 16b | Cite studies that might appear to meet the inclusion criteria, but which were excluded, and explain why they were excluded. | Page 8-9, lines 251-269 | Studies that met most but not all eligibility criteria were excluded, such as those providing bulk glioma data without subtype separation (e.g., astrocytoma, oligodendroglioma) or those presenting pathway-level enrichment results without reporting individual gene expression data. These were excluded to preserve dataset homogeneity and ensure gene-specific annotation |
| Study characteristics | 17 | Cite each included study and present its characteristics. | Page 8-9 | Characteristics of the included studies, including publication year, cohort size, data source (e.g., TCGA, CGGA, institutional RNA-seq), geographic origin, and analytical platform, are provided in Supplementary Table 1. All included studies analysed adult GBM patient samples, with 78% using RNA-seq and the remainder microarray or mixed platforms |
| Risk of bias in studies | 18 | Present assessments of risk of bias for each included study. |  | A formal risk of bias assessment tool (e.g., ROBINS-I) was not applied, as this synthesis integrated multiple transcriptomic datasets rather than interventional studies. However, bias risk was qualitatively considered based on cohort size (>50 patients considered lower bias risk), independent dataset validation, and clarity of reporting for analytical pipelines. |
| Results of individual studies | 19 | For all outcomes, present, for each study: (a) summary statistics for each group (where appropriate) and (b) an effect estimate and its precision (e.g. confidence/credible interval), ideally using structured tables or plots. | Page9-11 | Individual study-level gene expression values, prognostic associations, and targetability scores are summarised in Supplementary Tables 1-7, which provide numerical and categorical data for each gene alongside citations to the original source studies. |
| Results of syntheses | 20a | For each synthesis, briefly summarise the characteristics and risk of bias among contributing studies. | PAGE 9, LINE 255-259 | Across the 125 included studies, variation in analytical platform, patient cohort demographics, and endpoint definitions represented the primary sources of heterogeneity. The majority of datasets were derived from North American, European, or Chinese cohorts, with 58% cross-validated against TCGA or CGGA. Studies relying on single-institution datasets without external validation were considered at higher bias risk |
|  | 20b | Present results of all statistical syntheses conducted. If meta-analysis was done, present for each the summary estimate and its precision (e.g. confidence/credible interval) and measures of statistical heterogeneity. If comparing groups, describe the direction of the effect. |  | As no meta-analysis was conducted, pooled estimates, heterogeneity metrics, and sensitivity analyses were not applicable. Variation in study design and reporting precluded quantitative synthesis; therefore, findings are presented descriptively and supported by visual summaries. |
|  | 20c | Present results of all investigations of possible causes of heterogeneity among study results. |  | As no meta-analysis was conducted, pooled estimates, heterogeneity metrics, and sensitivity analyses were not applicable. Variation in study design and reporting precluded quantitative synthesis; therefore, findings are presented descriptively and supported by visual summaries. |
|  | 20d | Present results of all sensitivity analyses conducted to assess the robustness of the synthesized results. |  | As no meta-analysis was conducted, pooled estimates, heterogeneity metrics, and sensitivity analyses were not applicable. Variation in study design and reporting precluded quantitative synthesis; therefore, findings are presented descriptively and supported by visual summaries. |
| Reporting biases | 21 | Present assessments of risk of bias due to missing results (arising from reporting biases) for each synthesis assessed. | PAGE 8, LINE 238-240 | The possibility of reporting bias was addressed by including both high-impact and specialty journal publications, cross-checking study-reported gene lists against TCGA/CGGA data, and integrating findings from multiple geographic regions. |
| Certainty of evidence | 22 | Present assessments of certainty (or confidence) in the body of evidence for each outcome assessed. | PAGE 8 | Certainty in the body of evidence is moderate, strengthened by consistency of findings across multiple independent datasets, but limited by heterogeneity in transcriptomic platforms, patient populations, and incomplete reporting in some studies. |
| **DISCUSSION** | | |  |  |
| Discussion | 23a | Provide a general interpretation of the results in the context of other evidence. | PAGE 12-20 |  |
|  | 23b | Discuss any limitations of the evidence included in the review. | PAGE 21 |  |
|  | 23c | Discuss any limitations of the review processes used. | PAGE 21 |  |
|  | 23d | Discuss implications of the results for practice, policy, and future research. | PAGE 21 |  |
| **OTHER INFORMATION** | | |  |  |
| Registration and protocol | 24a | Provide registration information for the review, including register name and registration number, or state that the review was not registered. |  | *This review was not registered in a public database."* (You can add this sentence in the Methods → “Study Selection” or “Eligibility Criteria” section.) |
|  | 24b | Indicate where the review protocol can be accessed, or state that a protocol was not prepared. |  | A review protocol was not prepared prior to conducting this systematic review |
|  | 24c | Describe and explain any amendments to information provided at registration or in the protocol. |  | Since there’s no protocol, you can mark “Not applicable” here. |
| Support | 25 | Describe sources of financial or non-financial support for the review, and the role of the funders or sponsors in the review. | Page 22 | This research received no specific grant from any funding agency in the public, commercial, or not-for-profit sectors |
| Competing interests | 26 | Declare any competing interests of review authors. | Page 22 | *The authors declare no competing interests."* |
| Availability of data, code and other materials | 27 | Report which of the following are publicly available and where they can be found: template data collection forms; data extracted from included studies; data used for all analyses; analytic code; any other materials used in the review. |  | All data extracted from included studies, as well as the Python scripts used for figure generation, are available from the corresponding author upon reasonable request. Supplementary Tables provide the complete dataset of curated genes, functional assignments, and scoring matrices |

*As the checklist was provided upon initial submission, the page number/line number reported may be changed due to copyediting and may not be referable in the published version. In this case, the section/paragraph may be used as an alternative reference.
